# *Salmonella* Typhimurium, the major causative agent of foodborne illness inactivated by a phage lysis system provides effective protection against lethal challenge by induction of robust cell-mediated immune responses and activation of dendritic cells

**DOI:** 10.1186/s13567-017-0474-x

**Published:** 2017-10-25

**Authors:** Gayeon Won, John Hwa Lee

**Affiliations:** College of Veterinary Medicine, Chonbuk National University, Iksan Campus, Gobong-ro 79, Iksan, 54596 South Korea

## Abstract

*Salmonella* Typhimurium infection via foodborne transmission remains a major public health threat even in developed countries. Vaccines have been developed to reduce the disease burden at the pre-harvest stage, but the cell-mediated immune response against intracellular invasion of the pathogen is not sufficiently elicited by conventional killed *Salmonella* vaccines, which are safer than live vaccines. In this study, we developed a genetically inactivated vaccine candidate by introducing lysis plasmid pJHL454 harboring the λ phage holin–endolysin system into *S*. Typhimurium; we designated this vaccine JOL1950. In vitro expression of endolysin was validated by immunoblotting, and complete inactivation of JOL1950 cells was observed following 36 h of the lysis. Electron microscopic examinations by scanning electron microscopy and immunogold labeling transmission EM revealed conserved surface antigenic traits of the JOL1950 cells after lysis. An in vivo immunogenicity study in mice immunized with lysed cells showed significantly increased serum IgG, IgG1, and IgG2a levels. Further, we observed markedly increased in vitro cell proliferation and upregulation of Th1, Th2, and Th17 cytokines in the repulsed splenic T-cells of immunized mice. In dendritic cells (DCs) treated with lysed JOL1950, we observed a significant increase in dendritic cell activation, co-stimulatory molecule production, and levels of immunomodulatory cytokines. In addition, Th1 and Th17 cytokines were also released by naïve CD4^+^ T-cells pulsed with primed DCs. Lysed JOL1950 also protected against lethal challenge in immunized mice. Together, these results indicate that our vaccine candidate has great potential to induce cell-mediated immunity against *S*. Typhimurium by facilitating the activation of DCs.

## Introduction

Nontyphoidal salmonellosis (NTS) attributable to food-borne transmission poses a substantial public health challenge worldwide [1]. The incidence of NTS infection in the US is estimated to be 1 million annually, with 400 deaths [2]. *Salmonella enterica serovar* Typhimurium (*S*. Typhimurium) is one of the most common serotype causing NTS infections related to human illnesses, such as acute gastroenteritis [2]. Food can be contaminated with the pathogen during pre-harvest, harvest, and post-harvest periods. Severe invasive *Salmonella* infection such as bacteremia and septicemia frequently occurs in the immunocompromised population, resulting in hospitalization and death [3]. The ability of *Salmonella* to resist environmental stresses make it difficult to eradicate the pathogen in the food chain [4]. Over the last few years, cost-effective intervention measures to minimize the microbial load of raw products have been explored because the use of antibiotics has been compromised by the emergence of multidrug-resistant *Salmonella* [5, 6]. A recent systemic review reported that vaccination decreased *Salmonella* prevalence in market-weight finisher swine [7], which suggests that control measures at the farm level can reduce the risk of food-borne salmonellosis. Given the importance of a “farm-to-fork” approach for the control of zoonotic food-borne diseases [8], development of effective vaccine candidates against salmonellosis responsible for human illnesses could address public health concerns about zoonotic infection through consumption of contaminated animal meats.

Several experimental vaccines containing both inactivated and live attenuated candidates have been tested [9]. Particularly, auxotrophic mutants of *S*. Typhimurium have a potential as a live attenuated vaccine candidate against the infection [10, 11]. However, the immunomodulatory effects elicited by the live attenuated vaccines have not been sufficiently assessed; their immunogenicity was found to vary depending on injection route [12, 13]. New and efficient vaccine candidates that are easy to administer and that confer high immunogenicity are required to help protect against food-borne salmonellosis at the pre-harvest level.

Bacterial ghosts (BG) refer to the empty, non-living envelopes of Gram-negative bacteria produced by the action of the coliphage lysis gene [14]. Lysis gene expression facilitates formation of transmembrane tunnels on the surface of the bacteria through which cytoplasm and nucleic acids are expelled due to osmotic pressure. However, it has been reported that bacteria cell lysis mediated by gene *E* is not complete [15, 16], which has raised concerns about the safety of BG vaccines.

In the current study, to address this technical problem concerning the production of BG, we adapted a holin–endolysin component from bacteriophage λ to construct a genetically inactivated *S.* Typhimurium vaccine candidate. Holin and endolysin act in a cooperative manner to cleave peptidoglycan (PG) substrates of bacterial cell walls [17]. Endolysin, a cell wall-degrading enzyme, accumulates in the cytoplasm [18]. At a genetically predetermined time when the fatal membrane lesions were formed by holin, endolysin proteins escape through IM lesions and consecutively degrade the PG layers. We prepared the lysis plasmid pJHL464 harboring *S* and *R* genes encoding holin and endolysin, respectively, under convergent promoter components to prepare the novel inactivated *S*. Typhimurium vaccine strain JOL1950.

We analyzed whether the antigenic properties of the bacterial surface components were altered following endolysin-mediated lysis by immunogold labelling and found that the BG had an intact bacterial surface structure. Given that a conventional killed vaccine against salmonellosis failed to induce proper cell-mediated immunity (CMI) [19], which is required to defend against intracellular invasion and multiplication of the pathogen, we investigated the ability of our vaccine construct to be efficiently internalized by dendritic cells (DCs), which mediate T cell-related adaptive immunity [20]. Immunoregulatory effects and protection efficacy of the vaccine were further evaluated in vivo and in vitro. This work makes a vital contribution toward protecting against food-borne salmonellosis.

## Materials and methods

### Bacterial strains and plasmids

Bacterial strains and plasmid vectors utilized in this study are listed in Table 1. A balanced-lethal system based on the aspartate β-semialdehyde dehydrogenase (*asd*) gene was used to maintain the stability of the plasmid in an attenuated *Salmonella* strain [21]. The *asd* gene-deleted mutants were incubated with 50 μg/mL diaminopimelic acid (DAP) in culture media at 37 °C. The *asd* gene-deleted vaccine strain complemented by an *asd* ^+^ lysis plasmid (pJHL464) was grown in medium supplemented with 0.2% l-arabinose. All bacterial strains used in the study were stored at − 80 °C in Luria–Bertani (LB) broth with 20% glycerol.

**Table 1:** **Bacterial strains and plasmids utilized in this study**

Strain/plasmids	Description	References
*E. coli*		
JOL232 (χ6212)	F-λ-φ80 Δ(*lacZYA*-*argF*) *endA1 recA1 hadR17 deoR thi*-*1 glnV44 gyrA96 relA1 ΔasdA4*	Lab stock
*Salmonella* Typhimurium		
JOL 401	Wild type isolate from porcine, challenge strain	Lab stock
JOL1311	*Δasd*, used as base vaccine strain	Lab stock
JOL1950	JOL1311 containing pJHL464	This study
Plasmids		
pJHL172	*asd* ^+^ vector, pBR ori plasmid harboring cI857/λPR promoter, araC ParaBAD, phiX174 lysis gene *E*	[22]
pJHL319	T-easy vector harboring *E* gene ghost cassette with the convergent promoter system	This study
pJHL360	T-easy vector harboring the R ghost cassette with the convergent promoter system	This study
pJHL464	asd^+^ vector, pBR ori plasmid harboring cI857/λP_R_ promoter, *araC* P_*araBAD*_, the R ghost cassette composed of S, R and R1/Rz genes	This study

### Construction of the vaccine strain

The plasmid pJHL464 contains a 1.4 kb fragment of the R lysis cassette comprising open reading frames (ORF) of the *S* and *R* genes encoding holin and endolysin proteins, respectively, and overlapping ORF of the *Rz/Rz1* genes. Convergent promoter components, namely an arabinose-inducible promoter (ParaBAD) and thermo-sensitive λpR promoter with a cI857 repressor system, were used for stringent regulation of the expression of the lysis cassette in the plasmid. For the construction, the 1.4 kb lysis cassette was digested and subcloned into the NcoI/BamHI-digested pJHL319 plasmid which is a T-easy vector carrying the convergent promoter components. The lysis cassette was placed between an upstream λpR promoter and downstream anti-sense araBAD promoter controlling the sequential expression of holin and endolysin harbored in the resultant plasmid, pJHL360. The total 4.2-kb DNA fragment which harbors the R lysis cassette and the dual promoter components was inserted into BglII/XhoI digested *asd* ^+^plasmid pJHL172 [22]. The resultant plasmid pJHL464 was initially introduced into Δ*asd E. coli* JOL232 (χ6212) to overcome the instability of the plasmid. Subsequently, the resultant plasmid was transformed into Δ*asd S*. Typhimurium strain JOL1311 by electroporation. The constructed strain was designated JOL1950. Expression of endolysin was confirmed by Western blotting with rabbit hyperimmune sera against endolysin following the procedure described in a previous study [23]. Further, morphological changes of JOL1950 following the expression of lysis genes encoded by the pJHL464 plasmid were examined by scanning electron microscopy (SEM) [22].

### Lysis efficiency

The expression of endolysin is repressed below temperatures of 30 °C as a result of the cI857 protein binding to the operator region of the λpR promoter. Inactivation of thermo-labile repressor cI857 at temperatures above 40 °C results in strong expression of the lysis gene cassette. In the presence of arabinose, the anti-sense RNA produced by the ParaBAD promoter could bind to its complementary sense RNA of the lysis genes caused by the leaky λpR promoter, thus preventing the translation and expression of the lysis genes [24]. To examine whether the holin–endolysin system effectively inactivated JOL1950 cells, a single colony of JOL1950 was grown in LB broth supplemented with 0.2% l-arabinose to mid-log phase at 27 °C. The number of ghost cells were determined by measuring optical density (OD at 600 nm wavelength) when the cells were grown to mid-log phase under the condition repressing expression of endolysin. To generate JOL1950 ghost cells, the inoculation was resuspended with LB broth to remove the arabinose and then incubated at 42 °C in a shaking incubator (150 rpm). Lytic capacity was examined by determining the viability of cells recovered in the culture sampled under conditions activating expression of the R lysis cassette. Cell viability was confirmed by the number of colony forming units (CFU) in 100 μL of the cells following the lysis procedure spreading on LB plate containing 0.2% l-arabinose in triplicate. The number of living cells were counted on the plate using a 10-fold serial dilution method. After 36 h of lysis, a pellet of completely inactivated JOL1950 ghost cells was stored at − 70 °C until further processing.

### Immunogold labeling

Transmission electron microscopy (TEM) was conducted to clarify whether the expression of the R lysis cassette in JOL1950 affected its native antigenicity following the protocol described in a previous study [25] with slight modification. JOL1950 cells inactivated for 24 h were fixed with 2% paraformaldehyde and 2% glutaraldehyde in 0.1 M sodium cacodylate buffer (pH 7.4) overnight at 4 °C. Fixed cells were washed with phosphate-buffered saline (PBS) three times and then absorbed onto carbon formvar-coated copper grids (200-meshes) for 10 min. The cells placed onto the grid were blocked in PBS containing 2% BSA for 30 min and then incubated with polyclonal antibody produced against JOL1311 in chicken (1:300) as a primary antibody. Sera containing the chicken polyclonal antibody were obtained from a white leghorn chicken at 4 weeks of age intramuscularly immunized with 1 × 10^7^ CFU of JOL1311 cells twice within a 2-week interval. Serum samples were obtained 14 days after the final immunization and stored at − 20 °C until use. Subsequently, cells were placed on 10 nm gold-labeled goat anti-chicken IgY (1:500; Abcam Inc., USA) as a secondary antibody for 30 min. After rinsing with PBS three times, the grids were stained with 2% uranyl acetate and analyzed. JOL1311 cells chemically inactivated by 0.2% formalin were used as the control.

### Animal experiments

All animal experimentation work was approved by the Chonbuk National University Animal Ethics Committee (CBNU2015-00085) and was carried out according to the guidelines of the Korean Council on Animal Care and Korean Animal Protection Law, 2007; Article 13 (Experiments with Animals). Five-week-old female BALB/c mice (*n* = 16) were randomly assigned to two groups. Group A mice were inoculated with 10^8^ CFU of lysed JOL1950 cells in 100 μL of sterile PBS via the subcutaneous route at weeks 0 and 2, while mice in group B received only 100 μL of sterile PBS on the same schedule. Whole blood samples were collected from the retro-orbital sinus of mice at weeks 0, 2, 4, 6, and 8 post-inoculation (pi). Serum samples isolated from whole blood were stored at −80 °C until assayed by ELISA. Additional mice (*n* = 10) were subcutaneously inoculated with JOL1950 ghost cells (1 × 10^8^) and sacrificed for splenic-T cell analysis at week 2 pi. At 8 weeks following immunization, all mice were challenged with a lethal dose (1 × 10^6^) of a virulent *S*. Typhimurium strain, JOL401, via intraperitoneal administration. Mortality was observed in the injected mice for 11 days post-challenge.

### Assessment of antibody titers by enzyme-linked immunosorbant assay (ELISA)

Antibody responses specific to the *Salmonella* outer membrane protein (OMP) fraction were evaluated by an ELISA following the protocol previously reported [26]. The *Salmonella* OMP fraction prepared from the JOL401 strain [27] (500 ng/well) was coated onto ELISA microtiter plates (Greiner, Frickenhausen, Germany). Sera were diluted 1:100 for examination of IgG, IgG1, and IgG2a titers. The antibodies were detected with horseradish peroxidase (HRP)-labeled goat anti-mouse IgG, IgG1 and IgG2a, respectively (Southern Biotechnology, Birmingham, AL). Predicted serum IgG titers were determined directly from a standard curve based on serial dilutions of purified mouse immunoglobulins (Southern Biotechnology, Birmingham, AL, USA) [28]. Titers of serum IgG1 and IgG2a are presented as absorbance values at 470 nm.

### Antigen-specific splenic T cell-related immune response

#### Splenocyte preparation

Splenocytes were aseptically isolated from immunized and non-immunized mice at week 2 pi. The spleens sampled from the mice were mashed in PBS and filtered through a 70 μm cell strainer. The cells were suspended in RPMI 1640 medium (GIBCO, Cat. No. 11875093) containing 5% FCS (GIBCO, Cat. No. 10099141) and then incubated in a humidified 37 °C, 5% CO_2_ incubator overnight for further experiments.

#### In vitro cell proliferation assay

The proliferation response following in vitro antigen stimulation was determined by MTT colorimetric assay with 3-(4,5-dimethyl-thiazole-2-yl)-2,5-phenyltetrazolium bromide [29]. Murine splenocytes seeded in 96-well cell culture plates (1 × 10^6^ cells/well) were incubated with 20 μL of MTT (5 mg/mL) at 37 °C for 4 h, and then the culture supernatant was removed, followed by addition of 100 μL dimethyl sulfoxide (DMSO) to each well. Cell proliferation was determined by the absorbance at 570 nm of the water-soluble MTT formazan product.

#### Cytokine assay

To assess immunomodulatory cytokine profiles elicited by the vaccine candidate, mRNA expression of IL-2, IL-4, IL-8, IL-17, IL-23, and IFN-γ was measured in splenic lymphocytes pulsed with *Salmonella*-specific outer membrane protein (OMP) antigens. Splenocytes (1 × 10^6^ cells/well) were stimulated with 300 ng/mL of antigens in a 96-well plate and incubated at 37 °C in a 5% CO_2_ incubator for 48 h. Total RNA was isolated from the pulsed cell cultures using GeneAll® Hybrid-RTM (GeneAll Biotechnology, Seoul, Korea) following the manufacturer’s instructions. Subsequently, RNA samples were converted into cDNA with the ReverTra Ace® qPCR RT kit (FSQ-101, TOYOBO, Japan). The mRNA expression of cytokines was measured by performing real-time reverse transcription-polymerase chain reaction (RT-PCR) with SYBR® Green Real-time PCR Master Mix (QPK-201, TOYOBO, Japan). Primer pairs described in a previous study were used [30]. Transcript levels of each cytokine were determined using the relative fold change method (2^−(ΔΔCT)^) based on the expression of β-actin used as an internal standard, and results were compared between immunized and non-immunized groups [31].

#### Dendritic cell analysis

The ability of lysed JOL1950 cells to trigger a T-cell-related immune response was assessed by dendritic cell analysis. Differentiation of immature murine bone marrow-derived dendritic cells (BMDC) following in vitro infection with JOL1950 ghosts was assessed by measuring the expression of surface markers and release of immunomodulatory cytokines. In addition, to evaluating the capacity of mature DCs pulsed with JOL1950 to stimulate autologous naïve T-cells in vitro, expression of immunomodulatory T-cell cytokines was evaluated in CD4^+^ T-cells co-cultured with primed DCs via qRT-PCR. BMDC were aseptically isolated from female C57BL/6 mice at 6 weeks of age (*n* = 3) and cultured as described previously with slight modifications [32]. Briefly, 1 × 10^6^ cells were suspended in RPMI medium containing 10% FBS, penicillin (10 units/mL), streptomycin (10 μg/mL), l-glutamine (0.29 mg/mL), and cytokines (10 ng/mL murine GM–CSF and 5 ng/mL IL-4). On day 5 of culture, BMDC adhering to the bottom of the plate were seeded in six-well cell culture plates and then stimulated with either BG at a multiplicity of infection of 10 or LPS (500 ng/mL, serotype O127:B8, Sigma-Aldrich, St. Louis, MO, USA) as a positive control for 48 h. On day 7, the expression of co-stimulatory molecules such as CD40, CD80, and MHC class II on the differentiated BMDC was analyzed by fluorescence-activated cell sorting (FACS) with labeling of cells with FITC anti-mouse CD11c, APC anti-mouse CD40, APC anti-mouse CD80, and APC anti-mouse MHC class II (all Miltenyi Biotec) as described previously [33]. The population of DC expressing co-stimulatory surface markers was determined in CD11c-gated DC. Gene upregulation of cytokines related to DC differentiation such as IL-6, IL-10, IL-12, and TNF-α was assessed in stimulated BMDC by qRT-PCR [23]. The capacity of BMDC primed with ghosts to differentiate naïve CD4^+^ T cells into effector CD4^+^ T cells was further evaluated. Immature autologous CD4^+^ T cells were isolated from the splenocytes of female C57BL/6 mice at 6 weeks of age (*n* = 4) using a magnetic bead-based CD4^+^ T cell isolation kit (Miltenyi Biotec) according to the manufacturer’s instructions. Immature CD4^+^ T cells (1 × 10^8^) suspended in RPMI medium containing 10% FBS, penicillin (10 units/mL) and streptomycin (10 μg/mL) were co-cultured with 1 × 10^6^ of pulsed BMDC at 37 °C in a humidified 5% CO_2_ incubator for 24 h. Release of cytokines associated with differentiation and effector functions of Th1 and Th17 (IL-12, IL-17, IL-23, and IFN-γ) was measured by assessing transcript levels of these cytokines by qRT-PCR [23]. All primer sets used in the cytokine assay are described in previous studies [30, 34].

### Statistical analysis

Data were analyzed using GraphPad Prism 6 software (Graph Pad Software, Inc., San Diego, CA, USA). Non-parametric Mann–Whitney rank sum test was applied to determine the significance of differences between the immunized and non-immunized groups. A *P* value less than 0.05 was considered to be statistically significant. Data are presented as mean ± standard error (SE).

## Results

### Characterization of the vaccine construct JOL1950

**Figure 1:**
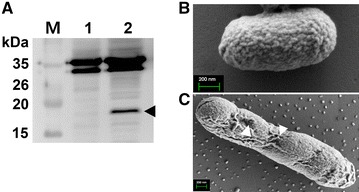
**Characterization of JOL1950 ghosts constructed by endolysin expression. A** Western blot analysis. Endolysin expressed in JOL1950 was confirmed by rabbit anti-endolysin polyclonal antibodies. Black arrows indicate the expected size of endolysin (~17 kDa). Lane M, size marker; lane 1, JOL1950 grown in the presence of l-arabinose at 27 °C; lane 2, JOL1950 ghosts produced under lysis conditions. Characterization of JOL1950 *S*. Typhimurium ghost cells by scanning electron microscopy (SEM). **B** Intact JOL1950 cells before lysis. **C** JOL1950 ghost cells after 36 h of lysis. Arrowheads indicate lysis transmembrane tunnels.

**Figure 2:**
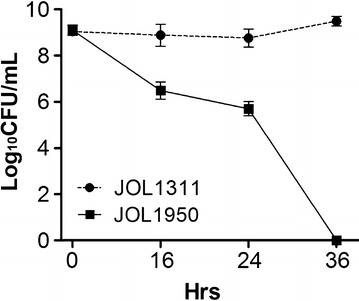
**Lysis pattern of** ***S*** **. Typhimurium harboring pJHL464 (JOL1950).** Cells grown to mid-logarithmic phase were inactivated by endolysin-mediated lysis. JOL1311 not carrying pJHL464 was used as the control. CFU counts were transformed to log base 10 values. Data are presented as mean ± standard error (SE) of three samples.

**Figure 3:**
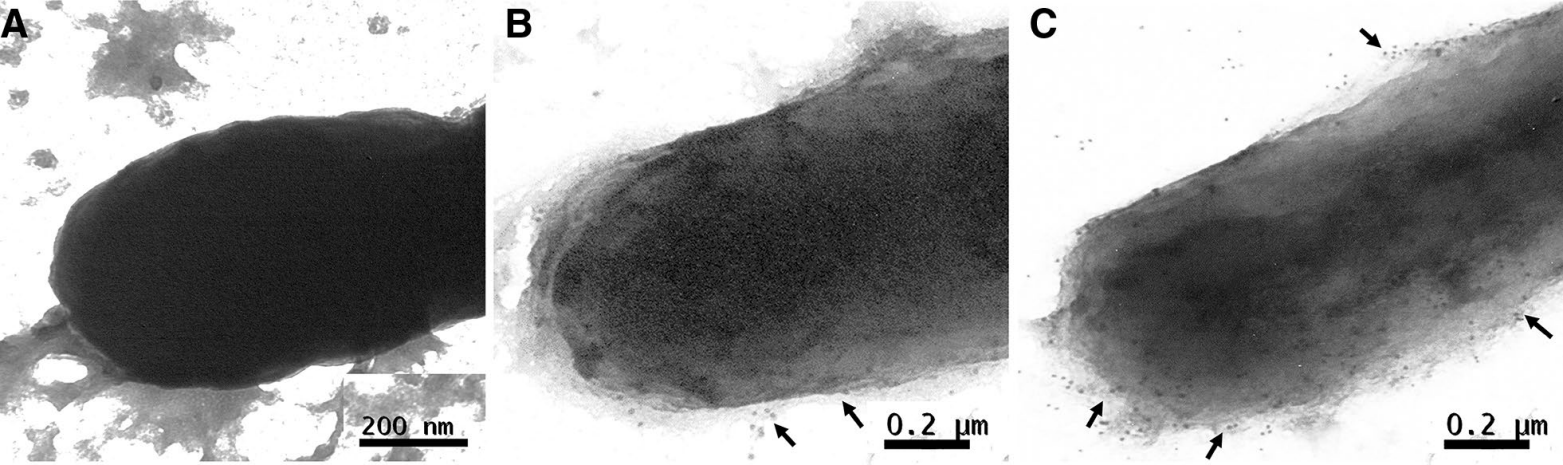
**Immunogold labelling of** ***S*** **. Typhimurium JOL1311 inactivated by chemical treatment or JOL1950 inactivated by endolysin expression.** Bacterial cells were immunogold-labeled and negatively stained for transmission electron microscopy. Arrows indicated the 10-nm gold particles binding the surface of cells. **A** Naïve JOL1311 cell, **B** JOL1311strain treated with 0.2% formalin, **C** *Salmonella* strain carrying pJHL464 inactivated by expression of lysis genes (JOL1950 ghosts).

In vitro expression of endolysin in JOL1950 was validated by Western blotting; a distinct immunoreactive band of ~ 17 kDa was found in lane 2 (Figure 1A). No band was detected in lane 1 (Figure 1A), which contained proteins extracted from cells grown under conditions repressing the activation of the lysis cassette. Morphological changes of JOL1950 cells after lysis were examined by SEM. After 36 h of lysis, the cell body was elongated, and the cell surface was partially shrunk due to inferred osmotic pressure across transmembrane tunnels (arrowheads, Figure 1C). Cell lysis efficiency of JOL1950 inactivated by the R lysis cassette was quantitatively compared with that of JOL1311 cells not harboring lysis genes. Cells were grown to mid-log phase in LB broth to which arabinose was added at 27 °C and were then washed with LB broth three times to remove the arabinose. Subsequently, washed cells were incubated in LB broth at 42 °C to induce the expression of the lysis genes. After 36 h of lysis, no viable cells were observed in the plate in which JOL1950 cells were inoculated (Figure 2). These results indicate that expression of the R lysis cassette in the JOL1950 was regulated by a transcriptional switch involving the convergent promoters, resulting in 100% lysis during the inactivation process. TEM analysis was used to assess possible alterations of antigenic properties on the bacterial surface. The results suggest that more antibody-bound colloidal gold particles may be bound to antigens on the surface of lysed JOL1950 compared to a chemically inactivated *Salmonella* strain (Figure 3). This finding implies that the structure of antigenic epitopes of the cells were less affected by the genetic inactivation strategy than chemical treatment.

### Assessment of total IgG isotypes and subclasses

**Figure 4:**
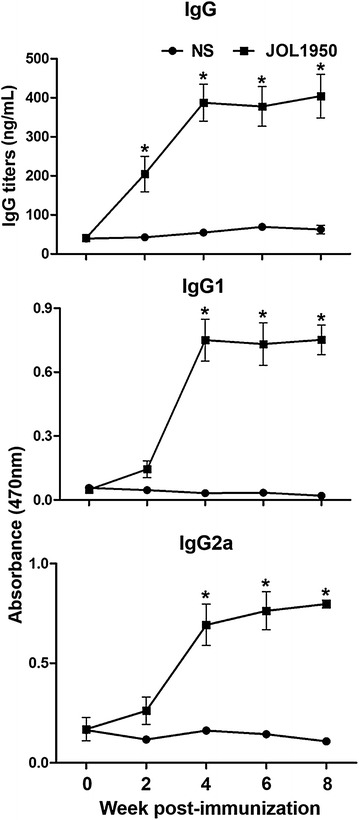
**Assessment of serum IgG antibody responses post-immunization.** Titers of serum IgG, IgG1, and IgG2a against a *Salmonella*-specific antigen OMP in immunized mice were measured by ELISA assay. Mice were subcutaneously injected with lysed JOL1950 while non-stimulated mice were inoculated with 100 μL of sterile PBS. Data are shown as mean ± standard error (SE). NS; non-stimulated, *P < 0.05, (vs. NS).

Antibody responses were assessed in mice immunized with JOL1950 ghosts. Levels of serum IgG specific to *Salmonella* OMP were markedly increased in the immunized group during the observational period after immunization compared to the control group (*P* < 0.05, Figure 4). The total concentration of the serum IgG subclasses IgG1 and IgG2a, which are specific markers of Th2 and Th1 cells, respectively [35], was also significantly augmented in immunized mice at weeks 4, 6, and 8 pi (*P* < 0.05, Figure 4), which demonstrated that JOL1950 immunization efficiently evoked activation of Th1- and Th2-related immune responses.

### Splenic T-cell proliferation assay

**Figure 5:**
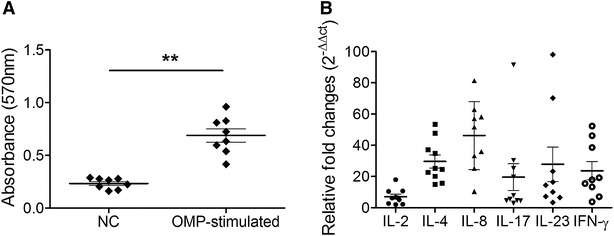
**T-cell immune responses elicited by JOL1950 immunization. A** Splenocyte proliferative responses against the OMP fraction in immunized (group A, *n* = 5) mice and non-immunized (group B = 5) mice which were used as a negative control (NC) at week 2 pi determined by absorbance values at 570 nm using the MTT assay. **B** Relative quantification (2^−∆∆Ct^) of cytokine mRNA levels in in vitro-stimulated splenic T cells isolated from mice in the immunized group. Data are presented as scatter plots and mean ± SE of five individual values in duplicate. **P < 0.001 (vs. restimulated NC).

Antigen recognition induces proliferation and differentiation of naïve T-cells into effector or memory T-cells, resulting in ensuing immune responses such as memory responses [36]. The magnitude of T-cell proliferation was assayed by the MTT colorimetric method in mice splenocytes re-pulsed in vitro with the OMP fraction. The proliferation response as assessed by absorbance at 570 nm was markedly increased (*P* < 0.001) in restimulated splenocytes isolated from immunized mice (0.668 ± 0.059) compared to those isolated from non-immunized mice (0.234 ± 0.018) (Figure 5A). This proliferation of splenic T cells in response to the antigen produced by lysed JOL1950 cells indicates the potential of this vaccine to induce cell-mediated immunity.

### Immunostimulatory cytokine analysis

Secretion profiles of immunomodulatory cytokines IL-2, IL-4, IL-8, IL-17, IL-23, and IFN-γ were analyzed by RT-PCR using RNA extracted from pulsed splenocytes isolated from immunized mice. Expression of cytokine genes associated with activation of CD4^+^ effector T helper (Th) cells in cell supernatants was compared to baseline expression (Figure 5B). At week 2 pi, there were 7.05 ± 1.54- and 23.73 ± 5.82-fold increases in mRNA levels of the Th1 cell associated cytokines IL-2 and IFN-γ, respectively. Immunization also upregulated mRNA expression of the Th2 cell-related cytokine IL-4 (29.67 ± 4.17-fold increase). mRNA expression of IL-17 and IL-23, which are cytokines expressed by Th17 cells [37], was increased by 19.65 ± 8.57-fold and 27.76 ± 11.08-fold, respectively.

### In vitro analysis of dendritic cells differentiated by the construct and indirect activation of naïve CD4^+^ T cells by the primed dendritic cells

**Figure 6:**
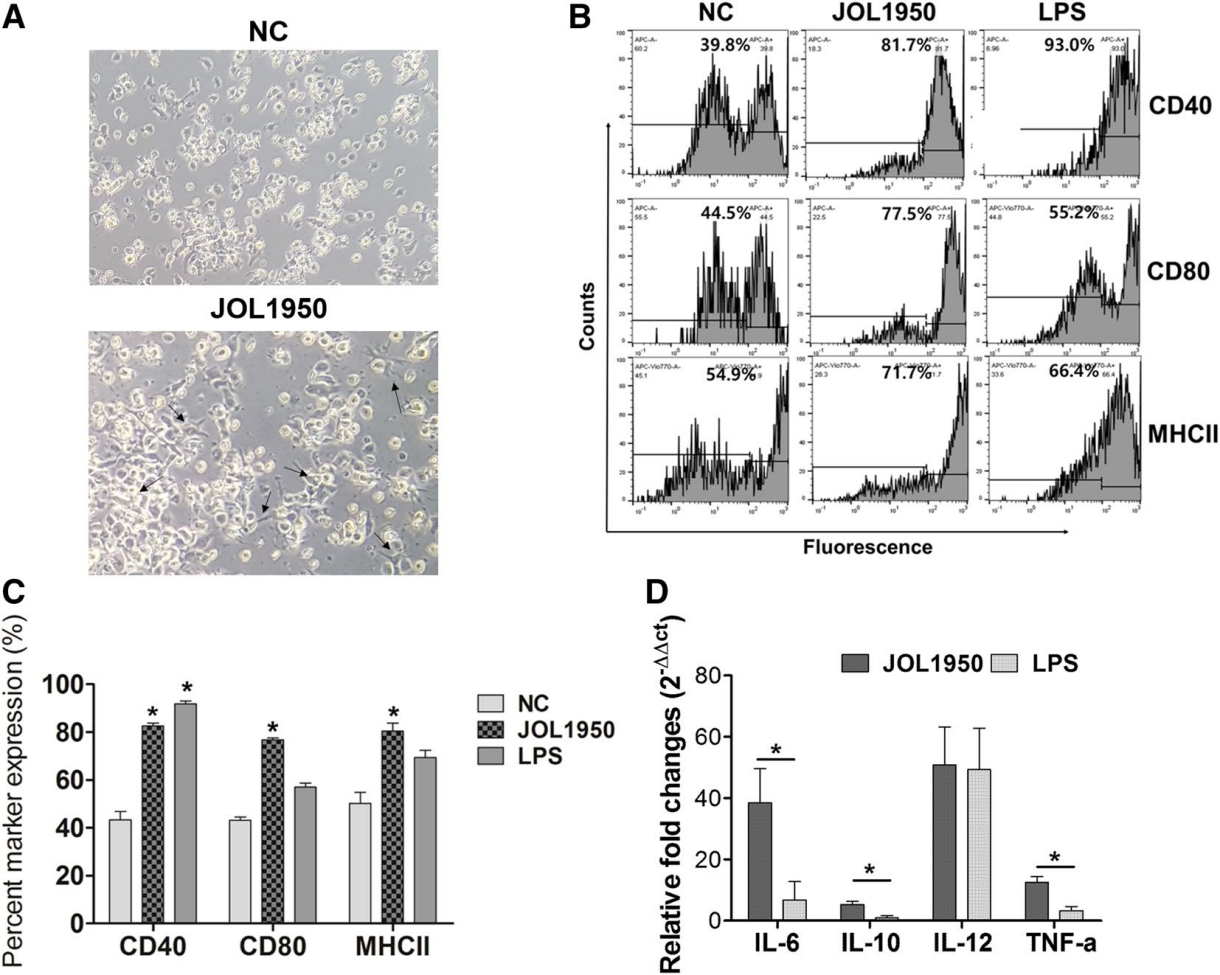
**In vitro analysis of dendritic cells differentiated by JOL1950 ghosts.** Co-stimulatory molecule expression on the dendritic cell surface was assessed by gating on CD11c^+^ cell populations by FACS. **A** Microscopic images of DCs captured with an inverted microscope (magnification × 100). Representative micrographs of DC culture medium containing murine GM–CSF and IL-4 during 5 days, and DC cultures stimulated with JOL1950 ghosts for an additional 48 h (on the 7th day of culture). DCs differentiated by stimulation displayed morphological characteristics typical of DCs, such as the presence of dendritic processes, as indicated by the arrows. DCs supplemented with GM-CSF and IL-4 or medium only were round in appearance or displayed veils. **B** Representative FACS histogram of the surface marker-positive cell population after 48 h of stimulation with the ghost cells or LPS. **C** Proportion (%) of co-stimulatory molecule-positive DCs after 48 h of stimulation with the ghost cells or LPS. *P < 0.05 (vs. non-stimulated DCs). **D** Cytokine mRNA upregulation in DCs co-cultured with BG. *P < 0.05 (vs. cytokine mRNA upregulated in LPS-stimulated DCs).

**Figure 7:**
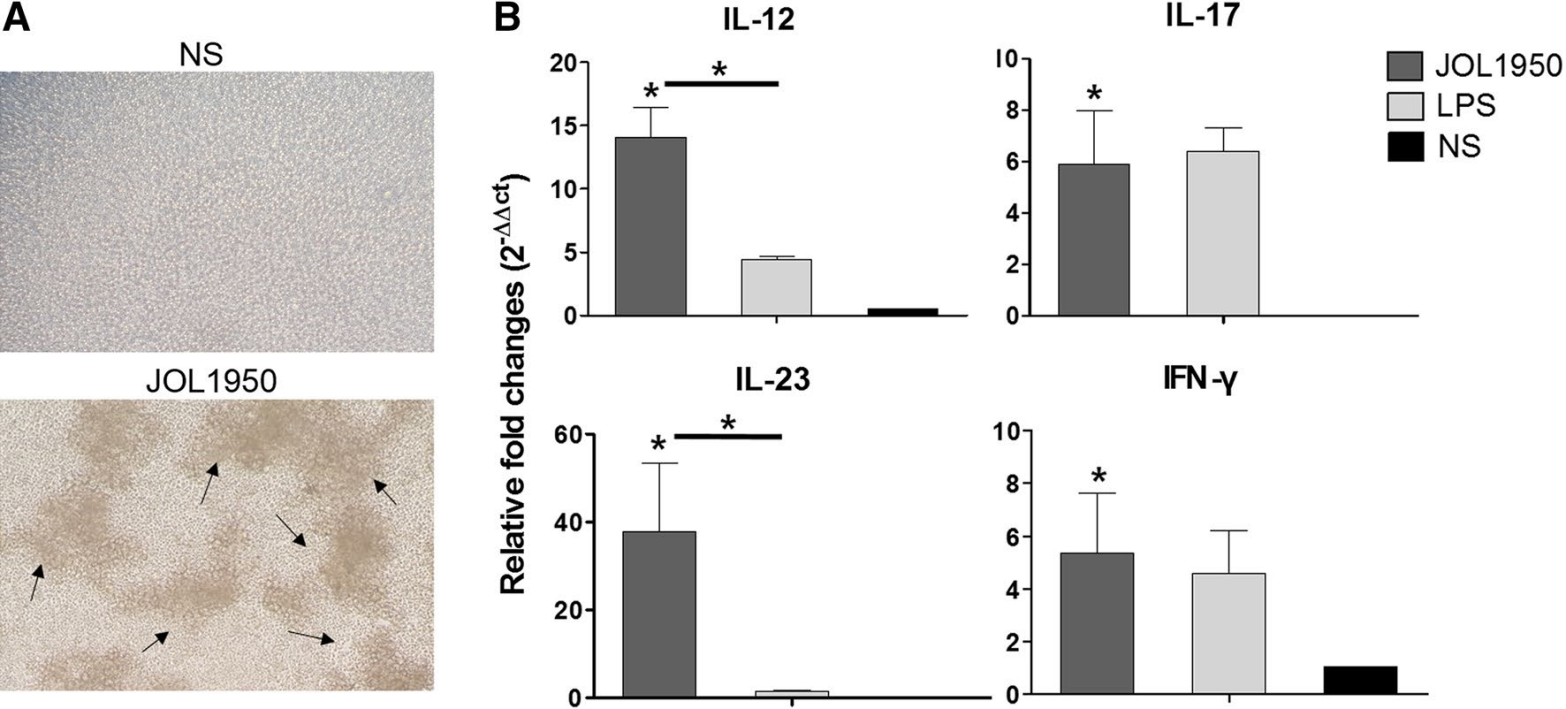
**Expression of T-cell-derived cytokines induced by indirect activation of naïve CD4** ^**+**^ **T cells by DCs primed with JOL1950 ghosts.** **A** Photographs of CD4^+^ T cells observed under an inverted microscope (magnification × 100). Culture of naïve CD4^+^ splenic T-cells and differentiated CD4^+^ T cells following stimulation with primed DCs co-infected with JOL1950 ghosts for 24 h. Accumulation of cells indicated by the arrows showed differentiation of cells due to stimulation. NS, non-stimulated. **B** Cytokine mRNA was upregulated in naïve CD4^+^ co-incubated with DCs primed with BG. Relative fold changes were calculated based on the 2^−ΔΔCT^ method. *P   <   0.05.

The capacity of JOL1950 ghosts to interact with BMDC to induce subsequent T-cell-related immunity was evaluated in vitro. After 48 h of stimulation with the ghost cells, distinctive morphological features such as large veils or multiple branches was shown on the surface of the DCs, typical signs of fully differentiated DCs (arrows, Figure 6A). The co-stimulatory surface markers MHC-II, CD40, and CD80 were markedly elevated in mature BMDC pulsed with ghost cells (Figures 6B and C). Lysed JOL1950 taken up by in vitro differentiated DCs elicited a significant increase in CD40, CD80, and MHC II production due to the presentation of antigenic peptides to T cells in primed DCs while the LPS stimulation only induced a marked increase in surface expression of CD40 on the DCs compared to non-primed DCs (*P* < 0.05). Differential induction of cytokines by DCs treated with lysed JOL1950 was observed. Cytokine mRNA levels were significantly upregulated in response to uptake of BG (Figure 6D). The mRNA expression of IL-12, which is vital for T-cell development [38], was increased in DCs co-cultured with ghosts or LPS. Transcript level of IL-6, a cytokine that stimulates naïve CD4^+^Th0 cells to differentiate into Th17 cells [39], was markedly upregulated in primed DCs (38.8 ± 11.3-fold increase) in comparison to DCs treated with LPS. We assessed the in vitro priming ability of DCs to evaluate whether DCs appropriately presented the processed antigen peptides to naïve CD4^+^ T cell in vitro and then efficiently activated CD4^+^ Th0 into effector T cells. The differentiated CD4^+^ T cells were observed after 24 h of stimulation with primed DCs co-incubated with JOL1950 ghosts (Figure 7A). Transcript levels of immunostimulatory Th1 cytokines (IL-12, IFN-γ) and Th17 cytokines (IL-17, IL-23) were significantly elevated in naive CD4^+^ T cells treated with pulsed DCs (Figure 7B). The significant increase in expression of cytokine genes indicated that lysed JOL1950 cells efficiently elicited the in vitro differentiation of CD4^+^ T cells into Th1 or Th17 phenotypes via activation of DCs.

### Lethal challenge

**Figure 8:**
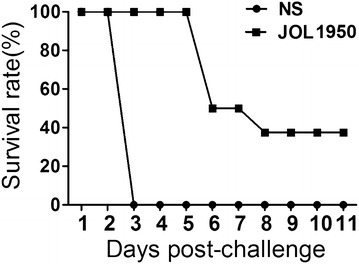
**Protective efficacy of JOL1950 ghost immunization against a lethal challenge with the virulent** ***S*** **. Typhimurium strain JOL401.** Survival rate of mice immunized with JOL1950 or PBS against intraperitoneal infection with a lethal dose of JOL401 is shown. Each group contained eight mice. NS, non-stimulated mice.

Protective efficacy of the *Salmonella* ghosts was evaluated by lethal challenge against *S*. Typhimurium infection. Mice were injected with a lethal dose of virulent *S.* Typhimurium JOL401 at week 8 pi. All control mice had died by day 3 post-infection; in contrast, all mice inoculated with JOL1954 were protected against the challenge by day 5 pi, and 3 of 8 mice survived to the end of the observation period (Figure 8).

## Discussion

*Salmonella* Typhimurium infection, which is mainly acquired through the uptake of contaminated food, can cause severe medical problems, particularly in immunocompromised individuals [40]. Vaccination is the most feasible tool to counteract infection and could be an effective pre-harvest intervention strategy to reduce fecal shedding of the pathogen [41]. In this study, we constructed a non-living *S*. Typhimurium vaccine candidate, JOL1950, which harbors the lysis plasmid pJHL464. Expression of endolysin under control of convergent promoters allowed stringent regulation of the transcriptional level of the lysis genes, as validated by both in vitro and in vivo studies (Figures 1 and 2). This strategy prevents leaky expression of lysis genes when cultivated under conditions repressing these genes, which is important for optimization of the mass production of a vaccine strain for large-scale clinical vaccine trials [22]. SEM analysis revealed that the JOL1950 ghosts retained their original cell morphology, but exhibited distinct surface wrinkles due to the loss of cytoplasmic contents via lysis tunnels around the cell body following the lysis procedure (Figure 1B). Immunogold labelling showed that JOL1950 ghosts constructed using the phage-lysis system conserved antigenic epitopes, resulting in higher surface antigenicity than a chemically inactivated *Salmonella* strain (Figure 3). Further, robust production of serum IgG specific to *Salmonella* OMP in immunized mice also demonstrated the ability of this vaccine to elicit antibody production against extracellular antigenic components of JOL1950 ghosts, which were not substantially altered during the endolysin-mediated lysis process (Figure 4). These results imply that the use of the holin–endolysin system resulted in successful production of an inactivated *Salmonella* vaccine, and that lysis did not negatively affect the surface antigenic characteristics of the resultant JOL1950 ghosts.

Vaccine selection against *Salmonella* is largely an empirical process due to a lack of understanding of how vaccine-induced immunity affects secondary intracellular infections. Thus, a promising vaccine candidate needs to induce cell-mediated immunity, as this is critical for the clearance of disseminated *Salmonella* infection. Conventionally killed *Salmonella* vaccines are known to induce good humoral immune responses, but poor Th1 type-cell responses [19]. Co-administration of a Th1-polarizing adjuvant chemical with a *Salmonella* killed vaccine was shown to help promote a Th1-related immune response, as assessed by lymphocyte proliferation [42]. In the current study, the inactivated vaccine candidate JOL1950 markedly increased proliferation of naïve splenic T-lymphocytes in response to antigen stimulation (Figure 5A). Enhanced serum levels of IgG2a and IgG1 subtypes as markers for Th1 and Th2 cells, respectively, were detected in immunized mice, indicating that immunization with lysed JOL1950 successfully drove the differentiation of naïve T cells into effector CD4^+^ T cell Th1 and Th2 subgroups. Concurrently, cytokines activating CD4^+^ Th1 T-cells and their effector cytokines, IL-2 and IFN-γ, were secreted by stimulated splenic lymphocytes. These findings indicated that the inactivated JOL1950 strain sufficiently induced cell-mediated immunity, which is known to accelerate the clearance of secondary *Salmonella* infection [19, 43].

Assessment of the ability of lysed JOL1950 cells to prime immature DCs, which are potent antigen-presenting cells, was used to evaluate the efficacy of the vaccine against *Salmonella* infection, since mature DCs differentiate by engulfing foreign proteins. It has been reported that BG produced by the expression of the PhiX174 lysis gene results in strong activation of DCs [44, 45]. Major antigenic components preserved on BG, which are referred to as pathogen-associated molecular patterns (PAMPs), are recognized by toll-like receptors (TLRs) and other pathogen recognition receptors (PRRs), resulting in activation of mature DCs and subsequent induction of T-cell-related immune responses. In this study, JOL1950 ghosts generated by the holin–endolysin lysis system efficiently triggered adaptive immune responses via DC-mediated antigen presentation. Expression of co-stimulatory molecules such as CD40, CD80, and MHC class II was significantly increased in murine BMDC stimulated with JOL1950 ghosts in comparison to naïve DCs (Figures 6B and C). The cytokine profile of DCs infected with ghost cells in vitro also demonstrated the immunostimulatory capacity of the JOL1950 ghosts (Figure 6C). Particularly, IL-12, which enhances T cell stimulatory capacity [46], was expressed by BMDCs pulsed with ghosts. The Th1-derived cytokines IL-12 and IFN-γ were produced by naïve CD4 cells cultured with primed BMDC. These results showed that PAMP derived from JOL1950 ghosts were properly detected by DCs, resulting in induction of T-cell-related CMI to combat intracellular *Salmonella* infection. Kirby et al. also reported that an immune response involving DCs and cytokine production is essential for controlling pathogen replication during the early stages of *Salmonella* infection [47]. Transcript levels of Th-17 effector cytokines IL-17 and IL23, which are involved in adaptive immunity, were also upregulated in CD4^+^ cells treated with pulsed DCs (Figure 7). Recent data demonstrated that intestinal Th17 cells play a pivotal role in engulfing pathogens that cross the epithelial mucosal barrier during the early phase of *Salmonella* infection [48]. Furthermore, immunization with JOL1950 effectively protected against a lethal dose of virulent *S*. Typhimurium (Figure 8). These findings demonstrate that JOL1950 inactivated by holin–endolysin system-mediated lysis induced differentiation of naïve T-cell into effector T-cells, which resulted in pathogen clearance through the production of cytokines by activated DCs.

Taken together, the present results that humoral and cellular immunity elicited by JOL1950 ghosts and their ability to protect against infection provide compelling evidence that JOL1950 ghosts are a promising avenue for generating inactivated vaccines against zoonotic salmonellosis. The immunization induced not only T-cell-mediated responses, but also host innate responses mediated by DCs that were activated by antigen recognition. Thus, given that conventional killed vaccines of intracellular *Salmonella* exhibit low and inconsistent protection efficacy, our genetically inactivated holin–endolysin phage lysis system represents a rational approach for development of an effective vaccine against foodborne salmonellosis.
